# Synthesis and Phase Transition of Poly(*N*-isopropylacrylamide)-Based Thermo-Sensitive Cyclic Brush Polymer

**DOI:** 10.3390/polym9070301

**Published:** 2017-07-23

**Authors:** Xiaoyan Tu, Chao Meng, Zhe Liu, Lu Sun, Xianshuo Zhang, Mingkui Zhang, Mingrui Sun, Liwei Ma, Mingzhu Liu, Hua Wei

**Affiliations:** State Key Laboratory of Applied Organic Chemistry, Key Laboratory of Nonferrous Metal Chemistry and Resources Utilization of Gansu Province, and College of Chemistry and Chemical Engineering, Lanzhou University, Lanzhou 730000, China; tuxy14@lzu.edu.cn (X.T.); mengch2013@lzu.edu.cn (C.M.); zhliu15@lzu.edu.cn (Z.L.); sunl2014@lzu.edu.cn (L.S.); zhangxsh16@lzu.edu.cn (X.Z.); zhangmk15@lzu.edu.cn (M.Z.); sunmr_14@lzu.edu.cn (M.S.); malw@lzu.edu.cn (L.M.)

**Keywords:** cyclic brush polymer, thermo-sensitivity, poly(*N*-isopropylacrylamide)

## Abstract

Polymers with advanced topological architectures are promising materials for wide applications due to their structure-generated unique properties different from that of the linear analogues. The elegant integration of stimuli-responsive polymers with such advanced architectures can create novel materials with virtues from both moieties, are thus a hot subject of research for both fundamental and practical investigations. To fabricate cyclic brush polymer-based intelligent materials for biomedical applications, herein, we designed and synthesized thermo-sensitive cyclic brush polymers with poly(*N*-isopropylacrylamide) (PNIPAAm) brushes by controlled living radical polymerization using cyclic multimacroinitiator. The thermo-induced phase transition behaviors of the resultant cyclic brush polymers with different compositions were investigated in detail by temperature-dependent optical transmittance measurements, and compared with the properties of bottlebrush and linear counterparts. Interestingly, the cloud point transition temperature (*T*_cp_) of cyclic brush PNIPAAm could be regulated by the chain length of PNIPAAm brush. Although the bottlebrush polymers with the same composition exhibited similarly structurally dependent *T*_cp_s behaviors to the cyclic brush polymers, the cyclic brush PNIPAAm did show higher critical aggregation concentration (CAC) and enhanced stability against dilution than the bottlebrush counterpart. The readily tailorable *T*_cp_s together with the ability to form highly stable nanoparticles makes thermo-sensitive cyclic brush PNIPAAm a promising candidate for controlled drug delivery.

## 1. Introduction

Polymers with advanced topologies, such as cyclic polymers, star-shaped polymers, bottlebrush polymers, and hyper-branched polymers, are promising materials for a broad range of applications due to their unique properties different from that of the traditionally linear analogues. Cyclic polymers with endless polymer chain architecture have been reported to result in advantages over linear counterparts with the same molecular weight (*M*_w_), including slower degradation behavior [1], compact structure with smaller size [1], enhanced fluorescent property [2], prolonged circulation time [3,4], improved drug loading and release capacity [5], and lower in vitro cytotoxicity [6]. Our group has a long-term interest in developing cyclic polymer-based and -derived materials for biomedical applications. The rapid advances in controlled living radical polymerization (CLRP) techniques [7], such as atom transfer radical polymerization (ATRP) [8] and reversible addition fragmentation chain transfer (RAFT) [9] polymerization, have witnessed the design and synthesis of well-defined polymers with advanced topologies, predetermined composition and narrow-distributed *M*_w_. In a recent study, we have developed a novel strategy towards cyclic brush polymer with precisely-controlled size by ATRP of a hydrophilic monomer using cyclic multimacroinitiator [10,11].

Traditionally amphiphilic copolymers are unable to sense a signal and respond by changing their structures. To develop stimuli-responsive polymers sensitive to environmental changes, the smart moiety was introduced into the polymer structure [12,13,14]. To date, numerous intelligent polymers, such as temperature [15,16,17], pH [18,19] and redox [20,21] responsive systems have been designed and developed for drug delivery applications. The elegant integration of stimuli-responsive polymers with the advanced architectures aforementioned can create novel materials with virtues from both moieties.

Temperature sensitivity is one of the most interesting properties in stimuli-responsive polymers. The most extensively investigated temperature sensitive polymer, poly(*N*-isopropylacrylamide) (PNIPAAm), exhibits a cloud point transition temperature (*T*_cp_) of approximately 33 °C in aqueous solution, below which PNIPAAm is water soluble and above which it becomes water insoluble [22,23] Basedon the thermo-sensitive property of PNIPAAm, many PNIPAAm-contained copolymers have been developed, which were utilized to prepare thermo-sensitive micelles as drug carriers [21]. In addition, it is worth pointing out that the *T*_cp_s of PNIPAAm-based polymers can be facilely tuned via copolymerization with hydrophilic or hydrophobic monomers [24].

Herein we reported the fabrication of PNIPAAm-based thermo-sensitive cyclic brush polymer. The effect of polymer topology on its thermo-induced phase transition was investigated in detail, and compared with that of the linear and bottlebrush analogues. Due to the readily tailorable *T*_cp_s and the highly stable nanoparticle formation, the cyclic brush PNIPAAm developed herein shows great potential as carriers for controlled drug delivery.

## 2. Materials and Methods

### 2.1. Materials

2-Hydroxyethyl methacrylate (HEMA, 99%, Sigma-Aldrich, Steinheim, Germany) was passed through a column filled with alkaline aluminum oxide to remove the inhibitor. *N*-isopropylacrylamide (NIPAAm, 99%, J&K, Beijing, China) was purified twice by recrystallization from *n*-hexane (97%, analytical grade, Rionlon, Tianjin, China). 2-bromoisobutyryl bromide (α-iBuBr, >98%, Sigma-Aldrich, Steinheim, Germany), 2,2′-bipyridyl (bpy, 98.0%, Sigma-Aldrich, Steinheim, Germany), *N,N,N*′,*N*″,*N*″-pentamethyldiethylenetriamine (PMDETA, 99%, Aladdin, Shanghai, China), copper(I) bromide (CuBr, >99.999%, Aldrich, Steinheim, Germany), sodium azide (NaN_3_, 98.0%, Sigma-Aldrich, Steinheim, Germany) were used as received. Tris[2-(dimethylamino)ethyl]amine (Me_6_TREN) was synthesized according to the reported procedure [25]. Anhydrous ethyl ether (99.0%, analytical grade, Rionlon, Tianjin, China), anhydrous methanol (99.5%, analytical grade, Rionlon, Tianjin, China), isopropyl alcohol (IPA, 99.7%, analytical grade, Kelong, Chengdu, China), *N*,*N’*-Dimethylformamide (DMF, 99.5%, analytical grade, Rionlon, Tianjin, China),pyridine (99.5%, XLHG, Guangdong, China) and other reagents were used as received.

### 2.2. Characterizations

#### 2.2.1. Proton Nuclear Magnetic Resonance Spectroscopy (^1^H NMR)

^1^H NMR spectra were recorded on a JNM-ECS 400 MHz spectrometer (JEOL, Tokyo, Japan operated in the Fourier transform mode. Dimethyl sulfoxide (DMSO-*d*_6_), and deuterated chloroform (CDCl_3_) were used as the solvents, respectively.

#### 2.2.2. Fourier Transform Infrared Spectroscopy (FT-IR)

The FT-IR spectroscopic measurements were conducted on a NEXUS 670 FT-IR spectrometer (Nicolet, WI, USA) and solid samples were pressed into potassium bromide (KBr) pellet prior to the measurements.

#### 2.2.3. Molecular Weight Determination

The size-exclusion chromatography and multi-angle laser light scattering (SEC-MALLS) analyses were used to determine the MW and molecular weight distribution (MWD, also termed as polydispersity (PDI)) of the polymers prepared. SEC using HPLC-grade DMF containing 0.1 wt % LiBr at 60 °C as the eluent at a flow rate of 1 mL/min, Tosoh TSK-GEL R-3000 and R-4000 columns (Tosoh Bioscience, Montgomeryville, PA, USA) were connected in series to a Agilent 1260 series (Agilent Technologies, Santa Clara, CA, USA), an interfero metricrefractometer (Optilab-rEX, Wyatt Technology, Santa Barbara, CA, USA) and a MALLS device (DAWN EOS, Wyatt Technology, Santa Barbara, CA, USA). The MALLS detector was operated at a laser wavelength of 690.0 nm.

#### 2.2.4. Ultraviolet-Visible Spectroscopy (UV–vis)

Temperature-dependent optical transmittance was examined on a Perkin-Elmer Lambda 35 UV–vis spectrometer (Perkin-Elmer, Waltham, MA, USA). A thermostatically controlled cuvette was employed, and the heating rate was 0.2 °C/min. The concentration of the polymer solution was 1 mg/mL using phosphate buffer saline (PBS, pH 7.4, 10 mM) as the solvent.

#### 2.2.5. Dynamic Light Scattering (DLS)

Average sizes of polymer self-assemblies prepared at various concentrations in PBS were determined by DLS on a Zetasizer (Nano ZS, Malvern, Worcestershire, UK) at a fixed detection angle of 90°. The polymer solution was passed through a Millipore 0.45 μm pore-sized syringe filter prior to measurements.

#### 2.2.6. Critical Aggregation Concentration (CAC) Determination

Fluorescence spectra were recorded on a LS55 luminescence spectrometer (Perkin-Elmer, Waltham, MA, USA). Pyrene was used as a hydrophobic fluorescent probe. Aliquots of pyrene solutions (2 × 10^−^^5^ M in acetone, 1 mL) were added to containers, and the acetone was allowed to evaporate. Ten milliliter aqueous polymer solutions at different concentrations were then added to the containers containing the pyrene residue. It should be noted that all the aqueous sample solutions contained excess pyrene residue at the same concentration of 2 × 10^−6^ M. The solutions were kept at room temperature for 24 h to reach the solubilization equilibrium of pyrene in the aqueous phase. Excitation was carried at 339 nm, and emission spectra were recorded ranging from 350 to 600 nm. Both excitation and emission bandwidths were 10 nm. From the pyrene emission spectra, the intensities (peak height) of the first (*I*_373_) and the second band (*I*_384_) were analyzed as a function of the polymer concentrations. A CAC value was determined from the intersection of the tangent to the curve at the inflection with the horizontal tangent through the points at low concentration.

### 2.3. Polymer Synthesis

#### 2.3.1. Synthesis of Linear PHEMA (*l*-Alkyne-PHEMA–Br)

*l*-alkyne-PHEMA–Br was prepared by ATRP of HEMA in a mixed solvent of IPA and DMF, using propargyl 2-bromoisobutyrate [6] as the initiator and bpy/CuBr as the catalyst. The molar ratio of monomer and initiator ([Monomer]/[Initiator]) was controlled at 50. Briefly, HEMA (1.47 mL, 11.8 mmol), initiator (48.3 mg, 0.236 mmol), and bpy (73.6 mg, 0.471 mmol) were dissolved in a 9:1 *w/w* % IPA/DMF mixture to obtain a 50% *w/w* HEMA solution. After three freeze-pump-thaw cycles, CuBr (relative molar ratio of initiator:CuBr:bpy = 1:1:2) was then added quickly under the protection of nitrogen flow. After another three freeze-pump-thaw cycles, the reaction mixture was sealed and placed in an oil bath preheated at 65 °C. The reaction mixture became viscous as the reaction progressed. After the reaction completed, the solution was quenched by exposing to the air. The reaction mixture was diluted with DMF, followed by precipitation in cold anhydrous diethyl ether to get the crude product. The crude product was purified by dialysis against distilled water to remove copper catalyst and any un-reacted monomer. The purified product was harvested by freeze-drying (1.09 g, yield 67.1%). ^1^H NMR (DMSO-*d*_6_, 400 MHz): δ0.61–1.04(m, –CCH_3_), 1.60–2.00 (b, –CH_2_C–), 3.58 (s, –CH_2_OH), 3.90 (s, –COOCH_2_–), 4.79 (s, –CH_2_OH).

#### 2.3.2. Synthesis of Azide End-Functionalized PHEMA (*l*-Alkyne-PHEMA–N_3_)

*l*-alkyne-PHEMA–N_3_ was synthesized by substitution reaction of *l*-alkyne-PHEMA–Br with NaN_3_. *l*-alkyne-PHEMA–Br (0.800 g, 0.123 mmol) and NaN_3_ in a 20-fold molar ratio excess were dissolved in a 1/4 *v/v* % water/DMF mixed solvent (10.0 mL) in a round-bottom flask. The flask was sealed and stirred in an oil bath preheated at 45 °C for 48 h. After purification by extensive dialysis to remove the residual sodium salts, *l*-alkyne-PHEMA–N_3_ was harvested by freeze-drying (0.720 g, yield 90.0%). ^1^H NMR (DMSO-*d*_6_, 400 MHz): δ 0.61–1.04 (m, –CCH_3_), 1.60–2.00 (b, –CH_2_C–), 3.58 (s, –CH_2_OH), 3.90 (s, –COOCH_2_–), 4.79 (s, –CH_2_OH).

#### 2.3.3. Synthesis of Cyclic PHEMA (*c*-PHEMA) by Intra-Chain Click Coupling Using *l*-Alkyne-PHEMA–N_3_ Linear Precursor

*c*-PHEMA was synthesized by the intra-chain click cyclization as reported previously [6,10,11]. In a typical procedure,750 mL of DMF was poured into a 1000 mL three-neck round flask and then degassed by nitrogen flow for 1 h at 100 °C. 20-fold molar equivalents of PMDETA and CuBr were then added into the flask. A solution of *l*-alkyne-PHEMA–N_3_ precursor (0.500 g, 0.0768 mmol) in degassed DMF (10.0 mL) was added to the flask *via* a syringe pump at a rate of 7 μL/min. After the complete addition of the polymer solution, the mixture was allowed to proceed for another 24 h. DMF was then removed under the reduced pressure, and the concentrated residue was transferred directly to a dialysis tube and dialyzed against distilled water to remove the copper catalyst. The resulting cyclic polymer, *c*-PHEMA, was obtained by freeze-drying (0.0700 g, yield 14.0%). ^1^H NMR (DMSO-*d*_6_, 400 MHz): δ 0.61–1.04 (m, –CCH_3_), 1.60–2.00 (b, –CH_2_C–), 3.58 (s, –CH_2_OH), 3.90 (s, –COOCH_2_–), 4.79 (s, –CH_2_OH).

#### 2.3.4. Synthesis of Cyclic Poly(2-Bromoisobutyrnyloxy)ethyl Methacrylate Multimacroinitiator (*c*-P(HEMA–Br)) and Linear Analogue *l*-P(HEMA–Br)

*c*-P(HEMA–Br)and *l*-P(HEMA–Br) were synthesized according to the literature [10,11,26]. Briefly, *c*-PHEMA (0.200 g, 1.537 mmol of HEMA units) was dissolved in 5 mL of dry pyridine. After cooling to 0 °C in an ice bath, 2-bromoisobutyryl bromide (1.00 mL, 7.70 mmol) was added dropwise to the rapidly stirring solution over 30 min. The solution was further stirred for 1 h at 0 °C, and then at room temperature for 24 h. At the end of the reaction, the mixture was added into the distilled water to precipitate the crude product. The product was separated by centrifugation, and purified twice by redissolving/reprecipitating with THF/water. The obtained product was dried under vacuum for 24 h (0.120 g, yield 60.0%). ^1^H NMR (DMSO-*d*_6_, 400 MHz): δ 0.61–1.04 (m, –CCH_3_), 1.90 (s, –COC(CH_3_)_2_Br), 1.70–2.00 (s, –CH_2_C–), 4.00–4.21 (s, –COOCH_2_CH_2_O–), 4.21–4.45 (s, –COOCH_2_CH_2_O–).

#### 2.3.5. Synthesis of Cyclic Brush and Bottlebrush Polymers (*cb*-P(HEMA-*g*-PNIPAAm) and *bb*-P(HEMA-*g*-PNIPAAm))

*cb*-P(HEMA-*g*-PNIPAAm) and *bb*-P(HEMA-*g*-PNIPAAm) with different degrees of polymerization (DPs) were prepared by ATRP using brominized cyclic and linear PHEMA as the multimacroinitiators, respectively, in a DMF/IPA mixed solvent. Taking the synthesis of *cb*-P(HEMA-*g*-PNIPAAm) as an example, *c*-P(HEMA–Br)(14.0 mg, 0.0500 mmol of initiator sites), NIPAAm (1.70 g, 0.0150 mmol) andMe_6_TREN(13.8 mg, 0.0600 mmol) were dissolved in a 1:1 *v/v* % DMF/IPA mixed solution (3.00 mL). After three freeze-pump-thaw cycles, CuBr (7.18 mg, 0.0500 mmol) was introduced under the protection of nitrogen flow. After another three freeze-pump-thaw cycles, the reaction mixture was sealed and placed in an oil bath thermostated at 60 °C. At the end of polymerization, the solution was quenched by exposure to air and diluted using THF. The crude product was collected by precipitation in excess ice-cold diethyl ether followed by purification by extensive dialysis against distilled water for 24 h to remove the copper catalyst. The resulting cyclic brush polymer was obtained by freeze-drying (0.0300 g, yield 17.7%). ^1^H NMR (CDCl_3_, 400 MHz): δ 0.61–1.04 (m, –CH(CH_3_)_2_), 2.00–2.50 (s, –CH_2_CH–), 3.95 (s, –NHCH(CH_3_)_2_), 5.80–6.90 (br, –CONHCH–).

*bb*-P(HEMA-*g*-PNIPAAm) was prepared following the same polymerization condition except using *l*-P(HEMA–Br) as the initiator.

## 3. Results and Discussion

### 3.1. Synthesis of PNIPAAm-Based Thermo-Sensitive Cyclic Brush Polymer

**Scheme 1:**
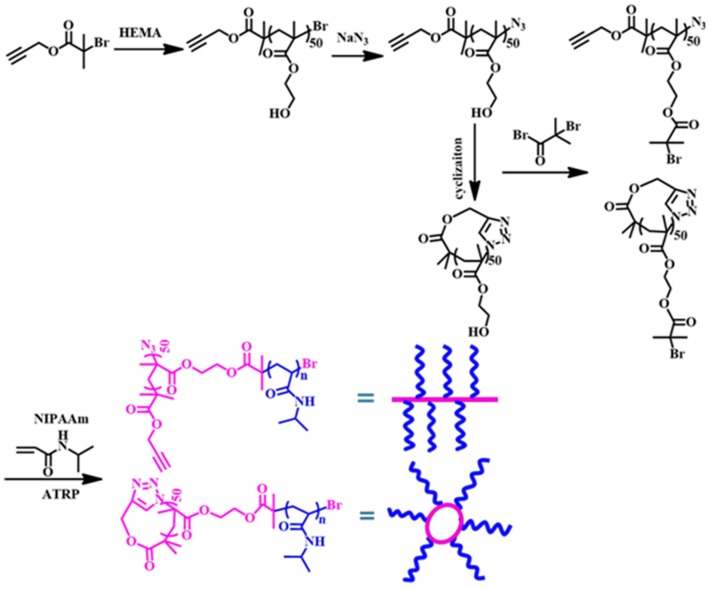
Synthesis of *cb*-P(HEMA-*g*-PNIPAAm) and *bb*-P(HEMA-*g*-PNIPAAm).

We have previously reported a strategy for synthesizing cyclic brush polymers using a combination of highly efficient click chemistry with ATRP grafting from technique [10,11,26], which was applied herein for the synthesis of cyclic brush polymers with thermo-sensitive brushes. The primary steps for the polymer synthesis are (a) synthesis of *l*-alkyne-PHEMA–Br by ATRP of HEMA using propargyl 2-bromoisobutyrate as the initiator; (b) nucleophilic substitution reaction of *l*-alkyne-PHEMA–Br with excess NaN_3_ to produce linear precursor, *l*-alkyne-PHEMA–N_3_; (c) cyclization of *l*-alkyne-PHEMA–N_3_ by intra-chain click coupling under extremely diluted conditions; (d) esterification of *c*-PHEMA with excess 2-bromoisobutyryl bromide to obtain *c*-P(HEMA–Br) multimacroinitiator; and (e)generation of target cyclic brush polymer, *cb*-P(HEMA-*g*-PNIPAAm) by ATRP of NIPAAm using *c*-P(HEMA–Br) as the multimacroinitiator and CuBr/Me_6_TREN as the catalyst. The synthesis of *cb*-P(HEMA-*g*-PNIPAAm) and *bb*-P(HEMA-*g*-PNIPAAm) was illustrated in Scheme 1.

In this study, thermo-sensitive cyclic brush polymers with two different DPs of PNIPAAm radiating rays were designed and synthesized using the same cyclic multimacroinitiator, *c*-P(HEMA–Br), with a fixed DP of 50, to investigate the effect of chain length of PNIPAAmgrafts on the thermo-induced phase transition of the corresponding cyclic brush polymers.

**Figure 1:**
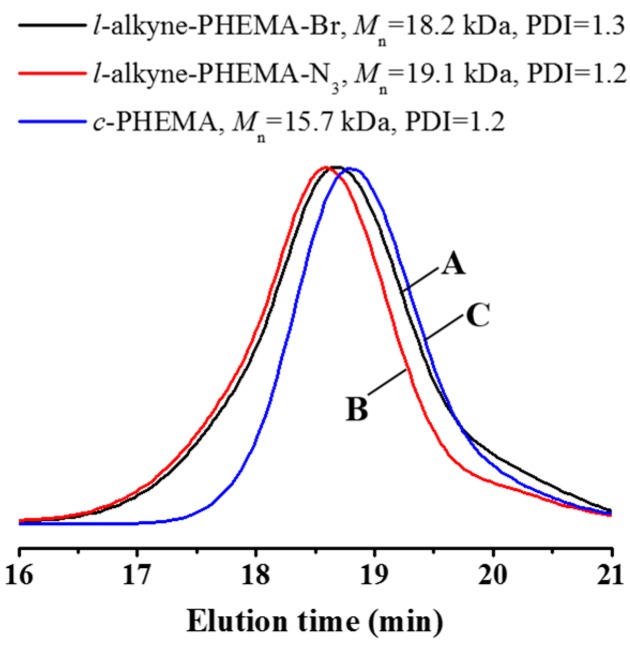
SEC elution traces of *l*-alkyne-PHEMA–Br (A); *l*-alkyne-PHEMA–N_3_ (B) and *c*-PHEMA (C) using DMF as the eluent.

^1^H NMR, FT-IR, and SEC-MALLS analyses were used to characterize the structure of the polymers synthesized. Specifically, the successful synthesis of cyclic PHEMA was confirmed by the disappearance of the characteristic band at ~2100 cm^−1^ assigned to the terminal azide group in the FT-IR spectrum after click coupling (Figure S1) as well as by a clear shift of the SEC elution trace towards lower MW after click cyclization compared to that of the linear precursor, *l*-alkyne-PHEMA–N_3_ (Figure 1). Both linear and cyclic polymers show uni-modal and narrowly distributed MW, demonstrating well controlled ATRP process and subsequent click coupling. More importantly, the <*G*> value of the cyclic polymer, defined as the apparent peak molar mass (*M*_p_) of the cyclic polymer to that of the linear precursor, was calculated to be ~0.92 for the *c*-PHEMA, which agrees well with the values reported for the other cyclic polymers (0.70–0.97) [27]. If inter-chain click coupling had taken place, polymer with multi-times MW would have been generated, and would have shown much larger <*G*> value. The results strongly support the successful preparation of *c*-PHEMA.

**Figure 2:**
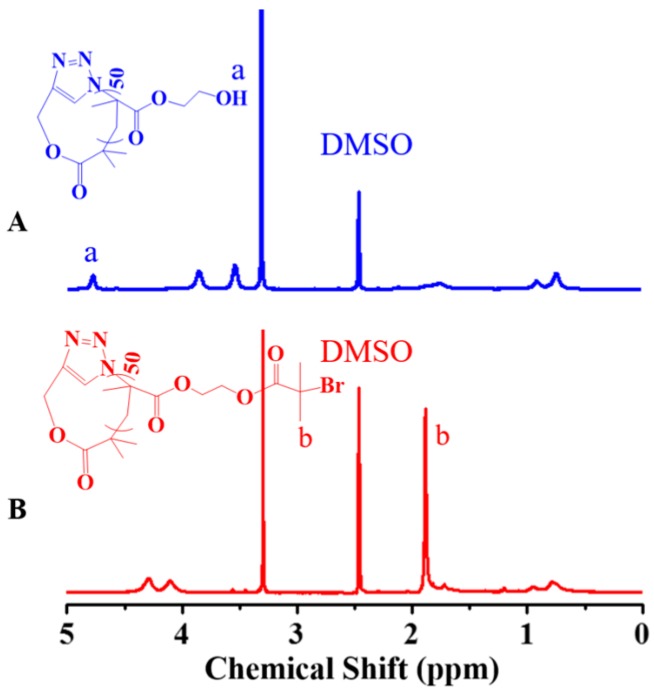
^1^HNMR spectra of *c*-PHEMA (**A**) and *c*-P(HEMA–Br) (**B**) in DMSO-*d*_6_.

**Figure 3:**
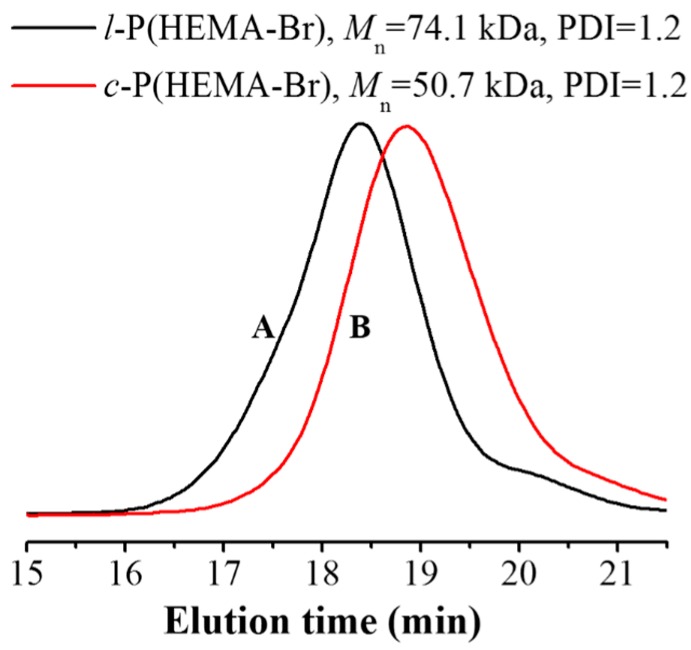
SEC elution traces of *l*-P(HEMA–Br)_50_ (A) and *c*-P(HEMA–Br)_50_ (B) using DMF as the eluent.

The multimacroinitiator, *c*-P(HEMA–Br) was then prepared by esterification between the hydroxyl group of HEMA unit and α-bromoisobutyryl bromide. Upon comparison of the ^1^H NMR spectra of *c*-PHEMA and *c*-P(HEMA–Br) (Figure 2),the disappearance of the characteristic signal at 4.79 ppm (band a), assigned to the hydroxyl group of HEMA units and the appearance of a strong resonance signal at 1.90 ppm (band b), attributable to the proton of methyl neighboring to the bromide after esterification reaction, confirm the complete conjugation of ATRP initiating sites to the pendant hydroxyl groups of the cyclic polymer. The *l*-alkyne-PHEMA–N_3_ was modified following the same strategy to produce *l*-P(HEMA–Br). *c*-P(HEMA–Br) and *l*-P(HEMA–Br)both show uni-modal and almost symmetric SEC elution peaks (Figure 3), indicating the nonoccurrence of any side reaction in the bromination step. The MWs, PDIs and DPs of linear and cyclic polymers synthesized were summarized in Table 1.

**Figure 4:**
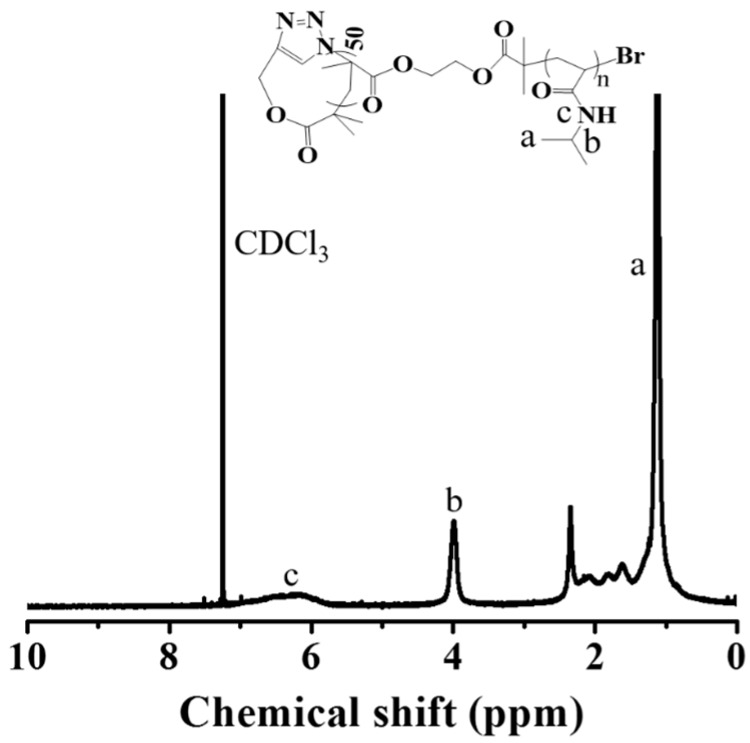
^1^H NMR spectrum of *cb*-P(HEMA-*g*-PNIPAAm)_50_ in CDCl_3_.

**Figure 5:**
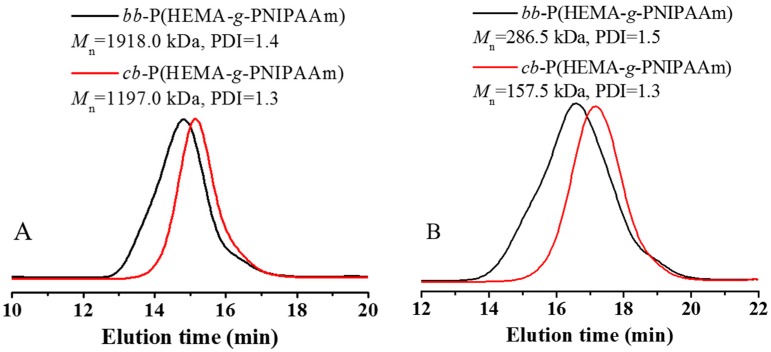
SEC elution traces of *bb*-P(HEMA-*g*-PNIPAAm_182_)_50_ and *cb*-P(HEMA-*g*-PNIPAAm_182_)_50_ (**A**) and *bb*-P(HEMA-*g*-PNIPAAm_42_)_50_ and *cb*-P(HEMA-*g*-PNIPAAm_42_)_50_ (**B**) using DMF as the eluent.

The thermo-sensitive cyclic brush and bottlebrush polymers with two different chain lengths of PNIPAAm graft brushes were prepared by ATRP of NIPAAm using *c*-P(HEMA–Br) and *l*-P(HEMA–Br) as the multimacro initiators, respectively. All the characteristic signals of PNIPAAm segment were recorded in the ^1^H NMR spectrum of *cb*-P(HEMA-*g*-PNIPAAm) (Figure 4), including the signal at 3.95 ppm (band “b”) assigned to the methenyl proton adjacent to the amide residues and the signal at 5.8–7.0 ppm (band “c”) ascribed to the secondary amine group, which confirms the successful initiation and subsequent propagation of PNIPAAm chains from the cyclic core template. Based on the conversion of NIPAAm calculated by ^1^H NMR analysis, the DP of PNIPAAm was calculated to be 182. The polymer was thus denoted *cb*-P(HEMA-*g*-PNIPAAm_182_)_50_. Both cyclic brush and bottlebrush polymers show narrowly distributed and symmetric SEC elution peaks (Figure 5), and no tailing or shoulder at the lower or higher molecule weight side could be discerned. The results indicate the absence of premature chain termination during the polymerization process. The MWs, PDIs and DPs of the polymers synthesized were summarized in Table 2. 

### 3.2. Thermal-Induced Phase Transition ofCyclic Brushand Bottlebrush Polymers

**Figure 6:**
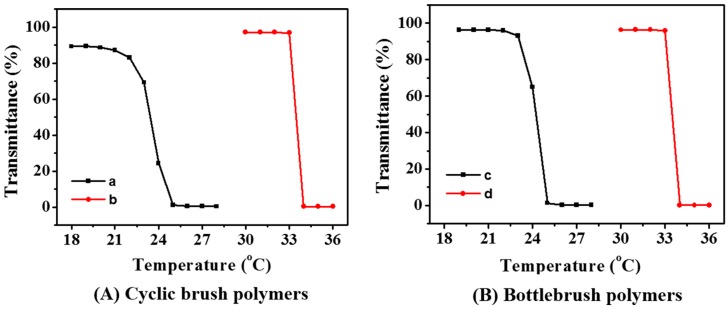
Temperature-dependent optical transmittance at 500 nm recorded for *cb*-P(HEMA-*g*-PNIPAAm_42_)_50_ (a); *cb*-P(HEMA-*g*-PNIPAAm_182_)_50_ (b); *bb*-P(HEMA-*g*-PNIPAAm_42_)_50_ (c) and *bb*-P(HEMA-*g*-PNIPAAm_182_)_50_ (d) (1.0 mg/mL, PBS, pH 7.4, 10 mM).

The thermal-induced phase transition temperature, also termed as *T*_cp_, is one of the basic physical properties of temperature-responsive polymers. Hydrogen bonding interaction of polymer with surrounding water molecules is the driving-force for the dissolution of PNIPAAm-type polymer in water, thus promoting the solubilization of PNIPAAm at room temperature, while the damage of hydrogen bonding above the *T*_cp_ due to the hydrophobic interactions contributes to its insolubilization [28,29]. The temperature-dependent transmittance of the *cb*-P(HEMA-*g*-PNIPAAm) and *bb*-P(HEMA-*g*-PNIPAAm) aqueous solutions were thus monitored by a UV/vis spectrometer equipped with a SHIMADZU temperature control system to investigate the phase transition behaviors. As shown in Figure 6, cyclic brush and bottlebrush polymers with the same chain length of PNIPAAm graft brushes show almost the same phase transition with identical *T*_cp_s at around 33 °C and 24 °C for PNIPAAm brushes with DPs of 182 and 42, respectively. The results suggest insignificant effect of polymer topology, i.e., cyclic brush VS bottlebrush structure, on the *T*_cp_ values. But note that there is a large difference between the *T*_cp_s of cyclic brush polymers with different chain lengths of PNIPAAm brushes, that is, the *T*_cp_ of *cb*-P(HEMA-*g*-PNIPAAm_182_)_50_ is very close to the one of pure PNIPAAm [30,31,32,33], but is significantly decreased to 24 °C for *cb*-P(HEMA-*g*-PNIPAAm_42_)_50_, so do the bottlebrush formulations. It has been reported that the *T*_cp_ depends primarily on the uninterrupted chain lengths of NIPAAm unit [34,35]. The cyclic brush polymer can be regarded as alternating copolymers composed of PNIPAAm graft brush and hydrophobic HEMA–Br unit, and the long continuous PNIPAAm chain was interrupted by the insertion of hydrophobic cyclic core, it is therefore reasonable to postulate that the *T*_cp_ of cyclic brush polymer was mainly determined by the repeated length of PNIPAAm graft brush. The brush with longer chain length than the cyclic core may be expected to have a *T*_cp_ similar to the one of pure PNIPAAm, but a significantly decreased *T*_cp_ was recorded for the polymers with short PNIPAAm brushes especially when the DP of brush is comparable to that of the cyclic core because copolymerization of NIPAAm with a hydrophobic monomer will shift the *T*_cp_ towards lower value [24].

Besides, the phase transition behaviors of cyclic brush and bottlebrush PNIPAAms can be also attributed to the confinement effects between the PNIPAAm chains, which is very similar to the results reported for the PNIPAAm brushes anchored on a solid substrate. This would be more apparent at the lower molecular weight than the high molecular weight because entropic freedom at high molecular weight would allow chains to avoid each other [36].

### 3.3. Nanoparticle Formation of Cyclic Brushand Bottlebrush Polymers in an Aqueous Solution

**Figure 7:**
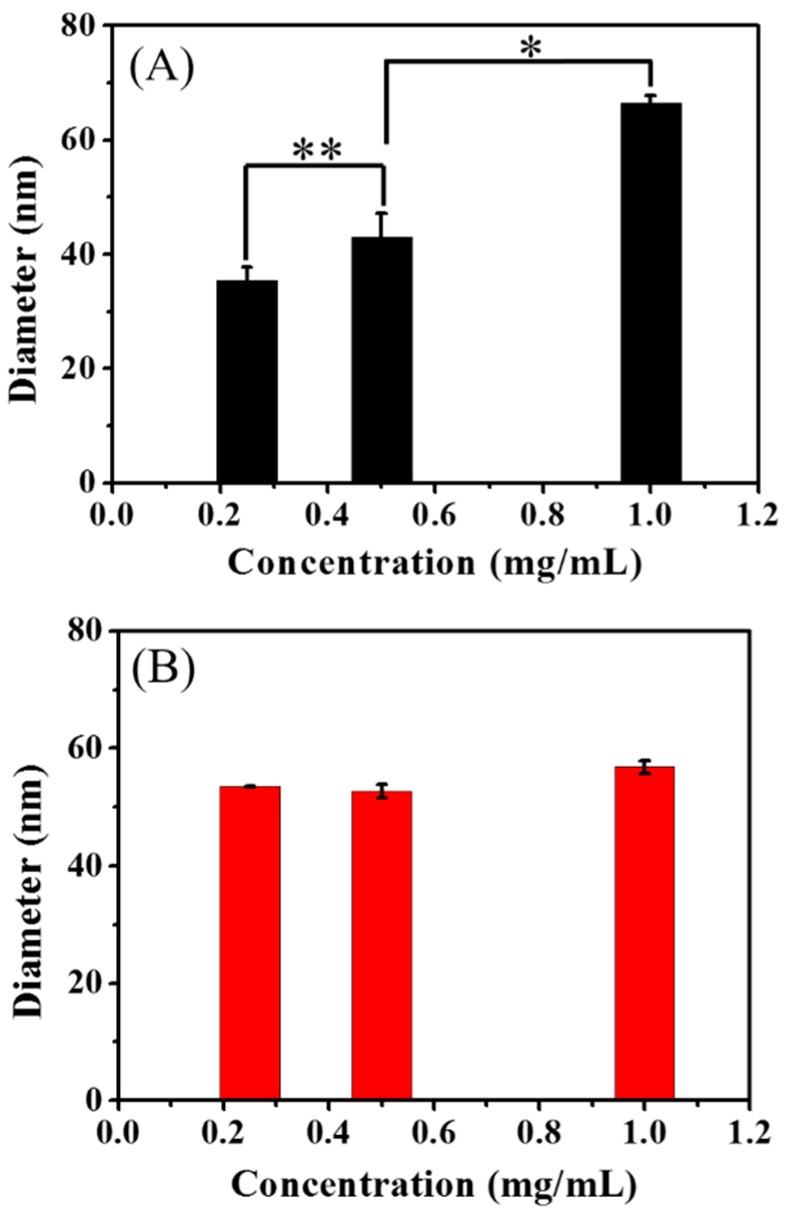
Average size of *bb*-P(HEMA-*g*-PNIPAAm_42_)_50_ (**A**) and *cb*-P(HEMA-*g*-PNIPAAm_42_)_50_ (**B**) at various concentrations determined by DLS at 25°C. (*p* refers to the confidence coefficient; *n* = 10; student’s *t*-test, * *p* < 0.001, ** *p* < 0.05).

The ability of the cyclic brush and bottlebrush polymers to form stable nanoparticles in deionized water was investigated by DLS measurements of polymer solutions at various concentrations of 1 mg/mL, 0.5 mg/mL, and 0.25 mg/mL (Figure 7). Interestingly, the average size of cyclic brush polymer keeps relatively constant around 50–60 nm irrespective of the dilution, which demonstrates the ability for the sunflower polymer to form nanoparticles with superior stability against dilution due to its hyper-branched cyclic brush structure. The size as well as the enhanced stability is highly desirable as controlled delivery systems to achieve prolonged circulation of nanocarriers, safe protection of cargoes encapsulated, and passive targeting by the so-called enhanced permeability and retention (EPR) effect. On the other hand, the bottlebrush polymer shows significantly decreased diameter from 67 nm at 1 mg/mL to 37 nm at 0.25 mg/mL, confirming the formation of aggregates at the high concentrations, and subsequent dissociation into unimers upon dilution. Taken together, we conclude it is the steric hindrance resulting from the densely grafted PNIPAAm brushes from the cyclic core that prevents the aggregation of sunflower PNIPAAm and contributes significantly to its formation of nanoparticles with enhanced stability.

To provide further an insight into the colloidal stability of nanoparticles formed by the cyclic brush and bottlebrush polymers, the CAC of both formulations were determined using pyrene as a fluorescence probe (Figure S2). The CAC of cyclic brush polymers (~240 mg/L) is clearly larger than that of bottlebrush polymer (~190 mg/L), which supports strongly the higher stability of nanoparticles formed by the cyclic brush polymers over that formed by the bottlebrush polymers, and agrees well with the DLS results.

## 4. Conclusions

In summary, thermo-sensitive cyclic brush polymers with PNIPAAm graft brushes were synthesized by ATRP of NIPAAm using cyclic multimacroinitiator. The thermo-induced phase transition behaviors of the resultant cyclic brush polymers with two different compositions were investigated and compared with the properties of bottlebrush and linear counterparts. The *T*_cp_s of cyclic brush and bottlebrush polymers are both dependent on the chain length of PNIPAAm graft brush due to the presence of a hydrophobic cyclic core or a linear backbone, i.e., long brush resulted in the polymers with an identical *T*_cp_ as pure PNIPAAm homopolymer, but a significantly decreased *T*_cp_ was recorded for the polymers with short PNIPAAm brushes. Moreover, the cyclic brush PNIPAAm did show enhanced stability than the bottlebrush counterpart due to its higher CAC value. The readily tailorable *T*_cp_ together with the capability to form highly stable nanoparticles thus makes thermo-sensitive cyclic brush PNIPAAm a promising candidate for controlled drug delivery. Further fabrication of thermo-sensitive cyclic brush polymers with a *T*_cp_s slightly above 37 °C by copolymerization of NIPAAm with a hydrophilic monomer is underway to realize hyperthermia-triggered drug delivery.

## Figures and Tables

**Table 1:** Summary of linear and cyclic polymers synthesized.

Sample	*M*_n_^a^ (kDa)	DP ^a^	*M*_n_^b^ (kDa)	PDI ^b^
*l*-alkyne-PHEMA_50_–Br	6.7	50	18.2	1.3
*l*-alkyne-PHEMA_50_–N3	6.7	50	19.1	1.2
*c*-PHEMA_50_	6.7	50	15.7	1.2
*l*-P(HEMA–Br)_50_	14.1	50	74.1	1.2
*c*-P(HEMA–Br)_50_	14.1	50	50.7	1.2

^a^ Determined by ^1^H NMR; ^b^ Determined by SEC-MALLS.

**Table 2:** Summary of *bb*-P(HEMA-*g*-PNIPAAm) and *cb*-P(HEMA-*g*-PNIPAAm) synthesized under different conditions.

Sample	Molar Feed Ratio of [NIPAAm]:[Br]	[NIPAAm](mol/L)	PolymerizationTime (h)	DP ^a^	*M*_n_^b^ (kDa)	PDI ^b^
*bb*-P(HEMA-*g*-PNIPAAm_182_)_50_	300	5	48	182	1918.0	1.4
*cb*-P(HEMA-*g*-PNIPAAm_182_)_50_	300	5	48	182	1197.0	1.3
*bb*-P(HEMA-*g*-PNIPAAm_42_)_50_	60	3	3	42	286.5	1.5
*cb*-P(HEMA-*g*-PNIPAAm_42_)_50_	60	3	3	42	157.5	1.3

^a^ Determined by ^1^H NMR; ^b^ Determined by SEC-MALLS.

## Supplementary Materials

The following are available online at www.mdpi.com/2073-4360/9/7/301/s1, Electronic supplementary information (ESI) available: FT-IR spectra of linear and cyclic polymers, and CAC determination of cyclic brush and bottlebrush polymers are available in Figures S1 and S2.
