# Supplementary material for: Synthesis and Phase Transition of Poly(N-isopropylacrylamide)-Based Thermo-Sensitive Cyclic Brush Polymer
Source: Polymers (Basel). 2017 Jul 23;9(7):301. doi: 10.3390/polym9070301 (PMC6432030; doi:10.3390/polym9070301)
Supplement: Supplementary file 1 [file polymers-09-00301-s001.pdf]

Supporting Information

of

Synthesis and Phase Transition of Poly(*N*-isopropylacrylamide)-based Thermo-sensitive Cyclic brush Polymer

1. **Figure S1**
2. **Figure S2**

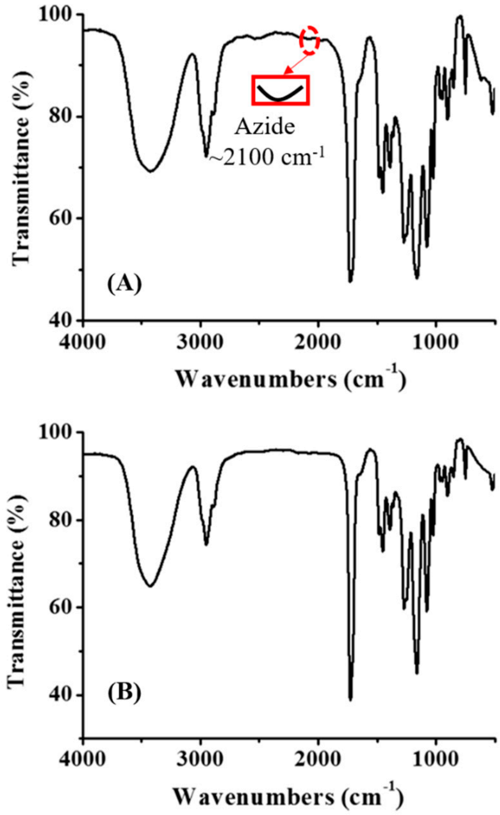

Figure S1. FT-IR spectra of *l*-alkyne-PHEMA-N_3 (A) and *c*-PHEMA(B).

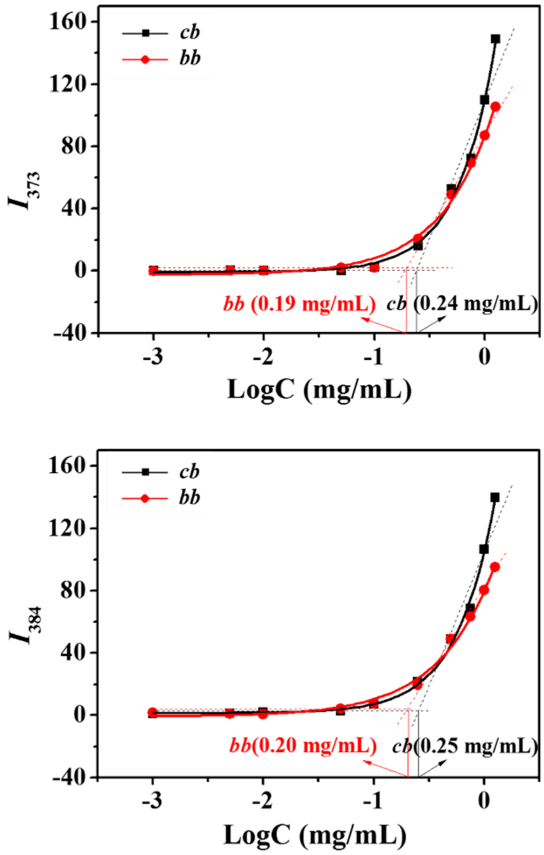

Figure S2. I_{373} and I_{384} in the emission spectra as a function of logarithm of *cb*-P(HEMA-g-PNIPAAm$_{182}$)$_{50}$ and *bb*-P(HEMA-g-PNIPAAm$_{182}$)$_{50}$ concentration. λ_{ex} = 339 nm. [Pyrene] =2 × 10 $^{-6}$ M.
